# Application of Minimal Physiologically-Based Pharmacokinetic Model to Simulate Lung and Trachea Exposure of Pyronaridine and Artesunate in Hamsters

**DOI:** 10.3390/pharmaceutics15030838

**Published:** 2023-03-03

**Authors:** Dong Wook Kang, Kyung Min Kim, Ju Hee Kim, Hea-Young Cho

**Affiliations:** College of Pharmacy, CHA University, 335 Pangyo-ro, Bundang-gu, Seongnam-si 13488, Gyeonggi-do, Republic of Korea

**Keywords:** pyronaridine, artesunate, minimal physiologically-based pharmacokinetic (PBPK) model, drug repurposing, COVID-19

## Abstract

A fixed-dose combination of pyronaridine and artesunate, one of the artemisinin-based combination therapies, has been used as a potent antimalarial treatment regimen. Recently, several studies have reported the antiviral effects of both drugs against severe acute respiratory syndrome coronavirus two (SARS-CoV-2). However, there are limited data on the pharmacokinetics (PKs), lung, and trachea exposures that could be correlated with the antiviral effects of pyronaridine and artesunate. The purpose of this study was to evaluate the pharmacokinetics, lung, and trachea distribution of pyronaridine, artesunate, and dihydroartemisinin (an active metabolite of artesunate) using a minimal physiologically-based pharmacokinetic (PBPK) model. The major target tissues for evaluating dose metrics are blood, lung, and trachea, and the nontarget tissues were lumped together into the rest of the body. The predictive performance of the minimal PBPK model was evaluated using visual inspection between observations and model predictions, (average) fold error, and sensitivity analysis. The developed PBPK models were applied for the multiple-dosing simulation of daily oral pyronaridine and artesunate. A steady state was reached about three to four days after the first dosing of pyronaridine and an accumulation ratio was calculated to be 1.8. However, the accumulation ratio of artesunate and dihydroartemisinin could not be calculated since the steady state of both compounds was not achieved by daily multiple dosing. The elimination half-life of pyronaridine and artesunate was estimated to be 19.8 and 0.4 h, respectively. Pyronaridine was extensively distributed to the lung and trachea with the lung-to-blood and trachea-to-blood concentration ratios (=C_avg,tissue_/C_avg,blood_) of 25.83 and 12.41 at the steady state, respectively. Also, the lung-to-blood and trachea-to-blood AUC ratios for artesunate (dihydroartemisinin) were calculated to be 3.34 (1.51) and 0.34 (0.15). The results of this study could provide a scientific basis for interpreting the dose–exposure–response relationship of pyronaridine and artesunate for COVID-19 drug repurposing.

## 1. Introduction

Drug repurposing is a rapid and safe method to determine a new usage of approved drugs in a cost-effective way [1]. In numerous cases, preclinical studies and clinical trials of repurposed drugs could be immediately started, thus accelerating drug approval with reduced time, costs, and risks [2]. The need for treatments against COVID-19 led researchers to utilize drug repurposing of approved drugs [3].

Artemisinin-based combination therapies (ACTs) are generally used to treat *Plasmodium falciparum* malaria, and World Health Organization (WHO) recommended ACTs as the first line of antimalarial therapy [4]. The fixed-dose combination of pyronaridine–artesunate which is one of the ACTs that was approved in Europe, Asia, and Africa for the treatment of uncomplicated malaria, and it is sold under the brand name Pyramax^®^ and Artecom^®^ with a 3:1 ratio of pyronaridine to artesunate. Recently, several articles have reported the antisevere acute respiratory syndrome coronavirus 2 (SARS-CoV-2) effect of antimalarial drugs including pyronaridine, artesunate, and dihydroartemisinin (the active metabolite of artesunate) in vitro and in vivo [5]. Gendrot et al. investigated the in vitro anti-SARS-CoV-2 activity of antimalarial drugs, and pyronaridine exhibited the most effective antiviral effect (EC_50_ = 0.72 μM; EC_90_ = 0.75 μM) [6]. Furthermore, the treatment of pyronaridine in SARS-CoV-2 infected mice showed significant inhibition of the viral load in the lungs and suggested that pyronaridine is a potential therapy for COVID-19 [7]. Cao et al. evaluated the in vitro anti-SARS-CoV-2 effect of nine compounds and artemisinin and showed the possibility of inhibiting SARS-CoV-2 replication in a dose-dependent manner [8]. In Call-3 cells, both pyronaridine and artesunate inhibited the growth and viral replication of SARS-CoV-2 and seasonal influenza A in a dose-dependent manner [9]. In addition, the combination therapy of pyronaridine–artesunate (including Pyramax^®^ and Artecom^®^) has been also investigated to evaluate the efficacy and safety of pyronaridine–artesunate in COVID-19 patients [10].

Lately, several researchers have utilized the physiologically-based pharmacokinetic (PBPK) model for drug repurposing of antiviral agents [11,12,13,14,15]. The PBPK model is a mathematical model describing absorption, distribution, metabolism, and excretion (ADME) based on physiological, physicochemical, and biochemical parameters [16]. The structure of the PBPK model generally consists of multiple tissue compartments and each compartment was connected by organ blood flow [17]. In addition, physiological parameters (cardiac output, tissue volume, etc.), tissue-to-blood partition coefficient, and biochemical parameters (metabolic clearance, renal or biliary clearance, protein binding rate, transporter activity, etc.) were required for developing the PBPK model [16]. The developed PBPK model could be used for predicting PKs, tissue distribution, excretion, etc., and it could provide a scientific basis for performing various extrapolations (species, routes, and dose levels) [18]. Although the PBPK modeling and simulation can provide reasonable predictions, the disadvantage of PBPK modeling is its complexity and accessibility; it depends on the quality of disposition data of the target drug (measurements of numerous organs and tissue concentrations are required) and due to their complexity, PBPK models are difficult to implement rapidly [19]. Due to the limitations of conventional PBPK models, minimal PBPK modeling was proposed as an alternative approach. In the minimal PBPK model, nontarget organs and tissues were lumped together for reducing the complexity of the conventional PBPK model. [20]. Thus, a detailed explanation of drug exposures for specific target tissues is possible.

Even if several articles have reported the in vitro or in vivo antiviral effects of pyronaridine and artesunate, there are limited data on the pharmacokinetics, lung, and trachea exposure that could be correlated with the antiviral effects. Thus, the purpose of the study was to evaluate the pharmacokinetics, lung, and trachea distribution of pyronaridine, artesunate, and dihydroartemisinin in golden hamsters for drug repurposing as an anti-SARS-CoV-2 treatment. The minimal PBPK models were developed and validated to predict the blood, lung, and trachea exposures of each compound, and the PBPK model was utilized for daily multiple-dosing simulation. The PK parameters, including elimination half-life, accumulation ratio, time to reach a steady state, and average blood, lung, and trachea concentration in the steady state, were evaluated for pyronaridine, artesunate, and dihydroartemisinin. The results of the study could be used as scientific evidence for interpreting the correlation between in vivo exposure and the anti-SARS-CoV-2 activity of pyronaridine and artesunate.

## 2. Materials and Methods

### 2.1. Chemicals and Reagents

Pyronaridine tetraphosphate (Lot No. PPNPGA008), artesunate (Lot No. AASTGA006), and dihydroartemisinin (Lot No. ICRS1410) were provided by Shin Poong Pharm. Co., Ltd. (Seoul, Republic of Korea). Amodiaquine, artemisinin, sodium phosphate tribasic dodecahydrate buffer, formic acid, ether, 85% ortho-phosphoric acid, ammonium acetate, and trifluoroacetic acid (TFA) were purchased from Sigma–Aldrich (St. Louis, MO, USA). Acetonitrile, methanol, water, and methyl-tert-butyl ether were purchased from J.T. Baker (Phillipsburg, NJ, USA). All other chemicals and reagents were HPLC or analytical grade.

### 2.2. Pharmacokinetic Study

#### 2.2.1. Animals

One hundred eight male golden hamsters were obtained from Janvier Lab. (Le Genest-Saint-Isle, France). All hamsters were maintained on a 12-h dark–light cycle at a temperature of 23 ± 3 °C and relative humidity of 55 ± 15%. This study was conducted according to the Guidelines for Ethical Conduct in the Care and Use of Animals and the rules of Good Laboratory Practice and was approved by the Institutional Animal Care and Use Committee (IACUC, protocol number SP2021-14) at Shin Poong Pharm. Co., Ltd. (Seoul, Republic of Korea).

#### 2.2.2. Study Design

The hamsters (102.01 ± 5.72 g) were divided into two groups: a low-dose group (*n* = 60) and a high-dose group (*n* = 48). In the low-dose group and high-dose group, 180/60 mg/kg and 360/120 mg/kg of pyronaridine/artesunate were administered orally once a day for 3 days to the hamsters. Blood samples (0.5 mL) were drawn from the jugular vein into heparinized tubes at the following times: 0 (predose), 0.08, 0.25, 0.5, 0.75, 1, 2, 4, 8, 12, 24, 47, 48.08, 48.25, 48.5, 48.75, 49, 50, 52, 56, 60, and 72 h during oral administration for 3 days. The blood sampling was conducted 1–3 times per hamster within 72 h, and the tissues were collected after the last blood sampling. The number of animals was four per blood and tissue sampling time point, respectively (each *n* = 4). The lung and trachea tissues were immediately obtained after sacrificing hamsters, washed in normal saline, and dried with filter papers. The lung and trachea sampling times were as follows: 0.08, 0.25, 0.5, 0.75, 1, 4, 8, 24, 48.08, 48.25, 48.75, 52, and 72 h. Tissues were individually weighed and homogenized with water (lung or trachea tissue: water = 1:4 *w*/*v*). The lung and trachea tissue homogenate were stored at −70 °C until sample analysis. For pyronaridine quantification, whole blood samples were stored at −70 °C until sample analysis. For quantifying artesunate and dihydroartemisinin, blood samples were centrifuged at 4 °C and 3000 rpm for 15 min followed by being transferred into clean tubes to obtain plasma and stored at −70 °C until sample analysis.

#### 2.2.3. LC-MS/MS Conditions

- Pyronaridine

Liquid chromatography was conducted on the Agilent 1290 Infinity II LC System (Agilent Technologies Inc., Santa Clara, CA, USA) coupled to a 6490 Triple Quad Mass Spectrometer (Agilent Technologies Inc., Santa Clara, CA, USA). A Synergi Max-RP column (2.0 × 75 mm, 4 μm particle size, Phenomenex, Torrance, CA, USA) was used at a temperature of 25 °C. The mobile phase consisted of 0.04% TFA (mobile phase A) and methanol:acetonitrile (3:1 *v*/*v*, mobile phase B) with a flow rate of 0.2 mL/min. A gradient elution was used for the chromatographic separation of pyronaridine as follows: 0.0–1.5 min, 10% B; 1.5–1.6 min, 10–60% B; 1.6–4.0 min, 60% B; 4.0–4.1 min, 60–10% B; 4.1–50 min, 10% B. The multiple reaction monitoring (MRM) mode was set with a positive electrospray ionization mode. The MRM transitions of pyronaridine and amodiaquine (internal standard, IS) were 518.2 > 447.1 and 356.2 > 283.0. The collision energy of pyronaridine and amodiaquine were 17 and 21 eV, and the cell accelerator voltage was 5 V for both compounds.

- Artesunate and dihydroartemisinin

The quantification of artesunate and dihydroartemisinin was conducted using the Nexera-X2 UPLC system (Shimadzu Corp., Tokyo, Japan) coupled with an LCMS-8040 mass spectrometer (Shimadzu Corp., Tokyo, Japan). Chromatographic separation was performed with an Inertsil ODS column (2.1 × 100 mm, 5 µm particle size, GL Sciences, Tokyo, Japan) column at a temperature of 25 ℃. The mobile phase consisted of 10 mM ammonium acetate (mobile phase A) and acetonitrile (mobile phase B) with an isocratic elution (A:B = 10:90, *v*/*v*), and a flow rate of 0.2 mL/min. The mass spectrometer was operated on electrospray ionization positive mode. MRM transitions were observed for artesunate (402.05 > 267.10), dihydroartemisinin (302.00 > 267.05), and artemisinin (IS, 299.95 > 283.15). The collision energy of artesunate, dihydroartemisinin, and artemisinin were 11, 9, and 7 eV.

#### 2.2.4. Sample Preparation

- Pyronaridine

Fifty μL of hamster blood, lung, or trachea tissue homogenates were added with 10 μL of the IS solution (10 μg/mL of amodiaquine). Added to the mixed sample were 125 μL of 0.5 M sodium phosphate tribasic dodecahydrate buffer (pH adjusted to 10.3 with 85% ortho-phosphoric acid) and 500 μL of ether, vortexed for 5 min and centrifuged at 21,130× *g* for 5 min. Then, 300 μL of supernatant were transferred to new microtubes and dried under nitrogen steam at room temperature. The dried residues were reconstituted with 100 μL of mobile phase (0.04% TFA:methanol:acetonitrile = 40:45:15, *v*/*v*/*v*) and vortexed for 5 min. After centrifugation at 21,130× *g* for 5 min, 5 μL of aliquot were injected into the UPLC-MS/MS system.

- Artesunate and dihydroartemisinin

Then, 50 μL of hamster plasma, lung, or trachea tissue homogenates were added with 10 μL of the IS solution (5 μg/mL of artemisinin). Next, 300 μL of acetonitrile were added to the mixture, vortexed for 5 min, and centrifuged at 21,130× *g* for 5 min. Then, 325 μL of supernatant were transferred to clean microtubes and dried under nitrogen steam at room temperature. The dried residues were reconstituted with 75 μL of mobile phase (10 mM ammonium acetate:acetonitrile = 10:90, *v*/*v*) and vortexed for 5 min. After centrifugation at 21,130× *g* for 5 min, 5 μL of supernatant was injected into the UPLC-MS/MS system. 

### 2.3. Development of Minimal Physiologically-Based Pharmacokinetic Models

The dispositions of pyronaridine and artesunate in hamsters were described by the minimal PBPK model and the PBPK modeling was performed using WinNonlin software (version 8.3, Certara™, Princeton, NJ, USA). A total of 530 measurements (343 for pyronaridine; 60 for artesunate; 127 for dihydroartemisinin) from 108 hamsters were used for the minimal PBPK modeling. The model consisted of five compartments including the absorption compartment, blood, lung, trachea, and the rest of the body. Also, nontarget tissues were lumped together into the rest of the body following the EPA guidance [16]. The equations describing the PKs, lung, and trachea distribution of pyronaridine were as follows:

- Minimal PBPK model for pyronaridine

$$\begin{array}{l} \frac{d\left(A_{a}\right)}{dt}=-\left(k_{a}\times A_{a}\right)\\ \frac{d\left(A_{blood}\right)}{dt}=\left(k_{a}\times A_{a}\right)+\left(\frac{C_{lung}\times Q_{co}}{K_{lung}}\right)+\left(\frac{C_{trachea}\times Q_{trachea}}{K_{trachea}}\right)+\left(\frac{C_{rest}\times Q_{rest}}{K_{rest}}\right)-\left(C_{blood}\times Q_{co}\right)\;-\left(C_{blood}\times Q_{trachea}\right)-\left(C_{blood}\times Q_{rest}\right)-\left(C_{blood}\times CL/F\right)\\ \frac{d\left(A_{lung}\right)}{dt}=\left(C_{blood}\times Q_{co}\right)-\left(\frac{C_{lung}\times Q_{co}}{K_{lung}}\right)+\left(A_{trachea}\times k_{tl}\right)-\left(A_{lung}\times k_{lt}\right)\\ \frac{d\left(A_{trachea}\right)}{dt}=\left(C_{blood}\times Q_{trachea}\right)-\left(\frac{C_{trachea}\times Q_{trachea}}{K_{trachea}}\right)+\left(A_{lung}\times k_{lt}\right)-\left(A_{trachea}\times k_{tl}\right)\\ \frac{d\left(A_{rest}\right)}{dt}=\left(C_{blood}\times Q_{rest}\right)-\left(\frac{C_{rest}\times Q_{rest}}{K_{rest}}\right)\\ C_{blood}=\frac{A_{blood}}{V_{blood}},C_{lung}=\frac{A_{lung}}{V_{lung}},C_{trachea}=\frac{A_{trachea}}{V_{trachea}} \end{array}$$

where *A_a_*, *A_blood_*, *A_lung_*, *A_trachea_*, and *A_rest_* are the amount of pyronaridine in the absorption compartment, blood, lung, trachea, and the rest of the body, respectively. *C_blood_*, *C_lung_*, and *C_trachea_* are blood, lung, and trachea concentrations of pyronaridine, respectively. *V_blood_*, *V_lung_*, and *V_trachea_* are the physiological volumes for blood, lungs, and tracheas of hamsters, respectively. *Q_co_* is the cardiac output of hamsters. *Q_trachea_* and *Q_rest_* are the blood flow rates for the trachea and the rest of the body, respectively. The first-order rate constants for absorption, trachea-to-lung transfer, and lung-to-trachea transfer are *k_a_*, *k_tl_*, and *k_lt_*. *K_lung_*, *K_trachea_*, and *K_rest_* are lung-to-blood, trachea-to-blood, and the rest of the body-to-blood partition coefficient, respectively. CL/F is the apparent total clearance of pyronaridine.

The minimal PBPK model was also developed for artesunate and its active metabolite (dihydroartemisinin). The model structure and parameters were similar to those of pyronaridine, and the parent PBPK model of artesunate was connected to the metabolite PBPK model of dihydroartemisinin as follows:

- Parent-metabolite PBPK model for artesunate

$$\begin{array}{l} \frac{d\left(A_{a}\right)}{dt}=-\left(k_{a}\times A_{a}\right)\\ \frac{d\left(A_{blood}\right)}{dt}=\left(k_{a}\times A_{a}\right)+\left(\frac{C_{lung}\times Q_{co}}{K_{lung}}\right)+\left(\frac{C_{trachea}\times Q_{trachea}}{K_{trachea}}\right)+\left(\frac{C_{rest}\times Q_{rest}}{K_{rest}}\right)-\left(C_{blood}\times Q_{co}\right)\;-\left(C_{blood}\times Q_{trachea}\right)-\left(C_{blood}\times Q_{rest}\right)-\left(C_{blood}\times CL/F\right)\\ \frac{d\left(A_{lung}\right)}{dt}=\left(C_{blood}\times Q_{co}\right)-\left(\frac{C_{lung}\times Q_{co}}{K_{lung}}\right)+\left(A_{trachea}\times k_{tl}\right)-\left(A_{lung}\times k_{lt}\right)\\ \frac{d\left(A_{trachea}\right)}{dt}=\left(C_{blood}\times Q_{trachea}\right)-\left(\frac{C_{trachea}\times Q_{trachea}}{K_{trachea}}\right)+\left(A_{lung}\times k_{lt}\right)-\left(A_{trachea}\times k_{tl}\right)\\ \frac{d\left(A_{rest}\right)}{dt}=\left(C_{blood}\times Q_{rest}\right)-\left(\frac{C_{rest}\times Q_{rest}}{K_{rest}}\right)\\ C_{plasma}=\frac{C_{blood}}{K_{b:p}}\;,\;C_{blood}=\frac{A_{blood}}{V_{blood}}\;,\;C_{lung}=\frac{A_{lung}}{V_{lung}}\;,\;C_{trachea}=\frac{A_{trachea}}{V_{trachea}} \end{array}$$

- Parent-metabolite PBPK model for dihydroartemisinin

$$\begin{array}{c} \frac{d\left(A_{blood,m}\right)}{dt}=\left(C_{blood}\times CL/F\right)+\left(\frac{C_{lung,m}\times Q_{co}}{K_{lung,m}}\right)+\left(\frac{C_{trachea,m}\times Q_{trachea}}{K_{trachea,m}}\right)+\left(\frac{C_{rest,m}\times Q_{rest}}{K_{rest,m}}\right)\\ -\left(C_{blood,m}\times Q_{co}\right)-\left(C_{blood,m}\times Q_{trachea}\right)-\left(C_{blood,m}\times Q_{rest}\right)-\left(C_{blood,m}\times CL_{m}/F\right)\\ \frac{d\left(A_{lung,m}\right)}{dt}=\left(C_{blood,m}\times Q_{co}\right)-\left(\frac{C_{lung,m}\times Q_{co}}{K_{lung,m}}\right)+\left(A_{trachea,m}\times k_{tl,m}\right)-\left(A_{lung,m}\times k_{lt,m}\right)\\ \frac{d\left(A_{trachea,m}\right)}{dt}=\left(C_{blood,m}\times Q_{trachea}\right)-\left(\frac{C_{lung,m}\times Q_{trachea}}{K_{lung,m}}\right)+\left(A_{lung,m}\times k_{lt,m}\right)-\left(A_{trachea,m}\times k_{tl,m}\right)\\ \frac{d\left(A_{rest,m}\right)}{dt}=\left(C_{blood,m}\times Q_{rest}\right)-\left(\frac{C_{rest,m}\times Q_{rest}}{K_{rest,m}}\right)\\ C_{plasma,m}=\frac{C_{blood,m}}{K_{b:p,m}}\;,\;C_{blood,m}=\frac{A_{blood,m}}{V_{blood}}\;,\;C_{lung,m}=\frac{A_{lung,m}}{V_{lung}}\;,\;C_{trachea,m}=\frac{A_{trachea}}{V_{trachea}} \end{array}$$

where *A_a_*, *A_blood_*, *A_lung_*, *A_trachea_*, and *A_rest_* are the amount of artesunate in the absorption compartment, blood, lung, trachea, and the rest of the body, respectively. *A_blood,m_*, *A_lung__,m_*, *A_trachea_*_,m_, and *A_rest,m_* are the amount of dihydroartemisinin in the blood, lung, trachea, and rest of the body, respectively. *C_plasma_*, *C_blood_*, *C_trachea_*, and *C_lung_* are plasma, blood, trachea, and lung concentrations of artesunate, respectively. *C_plasma,m_*, *C_blood,m_*, *C_trachea,m_*, and *C_lung,m_* are plasma, blood, trachea, and lung concentrations of dihydroartemisinin, respectively. *V_blood_*, *V_lung_*, and *V_trachea_* are the physiological volumes for blood, lung, and trachea of hamsters, respectively. *Q_co_* is the cardiac output of hamsters. *Q_trachea_* and *Q_rest_* are the blood flow rates for the trachea and the rest of the body, respectively. The first-order absorption rate constant is k_a_, and *k_tl_* and *k_lt_* are the first-order rate constants for trachea-to-lung and lung-to-trachea transfer of artesunate. The first-order rate constants for trachea-to-lung and lung-to-trachea transfer of dihydroartemisinin are *k_tl,m_* and *k_lt,m_*. *K_b:p_*, *K_lung_*, *K_trachea_* and *K_rest_* are blood-to-plasma, lung-to-blood, trachea-to-blood, and the rest of the body-to-blood partition coefficient for artesunate, respectively. *K_b:p,m_*, *K_lung,m_*, *K_trachea,m_* and *K_rest,m_* are blood-to-plasma, lung-to-blood, trachea-to-blood, and the rest of the body-to-blood partition coefficient for dihydroartemisinin, respectively. CL/F and CL_m_/F are the apparent total clearance of artesunate and dihydroartemisinin, respectively.

**Figure 1:**
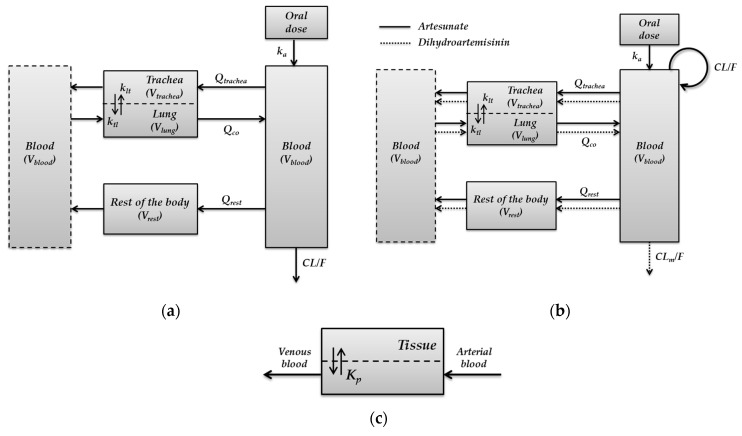
Structure of the minimal PBPK model for (**a**) pyronaridine, (**b**) artesunate, and dihydroartemisinin in hamsters. (**c**) The perfusion rate-limited kinetics was used to describe the equilibrium between blood and tissue concentrations.

The structures of the minimal PBPK models for pyronaridine and artesunate are shown in Figure 1a and 1b. In the minimal PBPK models of both pyronaridine and artesunate, a perfusion rate-limited kinetics with tissue-to-blood partition coefficient [21] was used to describe the equilibrium between blood and tissue concentrations of each drug (Figure 1c).

The physiological parameters of hamsters that were used to develop the minimal PBPK models of pyronaridine and artesunate are represented in Table 1. The blood volume (*V_blood_*) and cardiac output (*Q_co_*) of hamsters that were reported in the literature were used for the PBPK model development [22,23]. The blood flow rate for the trachea (*Q_trachea_*) was calculated by multiplying *Q_co_* by 2.1% [24,25,26]. The blood flow rate for the rest of the body (*Q_rest_*) was calculated by subtracting *Q_trachea_* from *Q_co_*. The average lung and trachea volumes (*V_lung_* and *V_trachea_*) were calculated from the individual lung and trachea weights of hamsters assuming the unit density (1 mL = 1 g) [16]. The volume of the rest of the body (*V_rest_*) was calculated by subtracting blood volume (*V_blood_*), lung volume (*V_lung_*), and trachea volume (*V_trachea_*) from the total body volume (102.01 mL) of the hamsters. Also, the reported values of blood-to-plasma partition coefficients for artesunate (*K_b:p_*) and dihydroartemisinin (*K_b:p,m_*) were used to calculate the blood concentrations from the plasma concentrations of both compounds. All physiological parameters were fixed, and biochemical parameters were fitted to develop the minimal PBPK model. 

A sensitivity analysis was performed for a quantitative evaluation of how model parameters (input parameters) influence the model predictions [16,17]. In this study, a normalized sensitivity analysis was conducted on the minimal PBPK models of pyronaridine and artesunate. The influence of model parameters on predicted blood concentrations was evaluated by using the *C_max_* and AUC resulting from increasing each parameter by 1%. The normalized sensitivity coefficients were calculated by original and changed parameters as the following equations [27,28]:

$$Normalized\;sensitivity\;coefficient=\frac{\frac{A-B}{B}}{\frac{C-D}{D}}$$

where *A* is the AUC calculated from the 1% increase in the biochemical parameter, *B* is the *C_max_* and AUC calculated from the original biochemical parameter, *C* is the increased biochemical parameter by 1%, and *D* is the original biochemical parameter. The normalized sensitivity coefficient of 1 means a 1:1 relationship between parameter changes and dose metric. If the normalized sensitivity coefficient was calculated to be 2, a 1% change in the input parameter results in a 2% change in *C_max_* or AUC. The normalized sensitivity coefficients were calculated from all biochemical parameters and those higher than 1 were considered to amplify the input error [16,29].

Also, the PK parameters (*C_max_*, AUC_Day1_, and AUC_Day3_) estimated from the observed and predicted concentrations of the three compounds were compared for evaluating overall model performance and calculating a fold error (FE) and average fold error (AFE) as follows [30,31,32,33]:

$$Fold\;error\;\left(FE\right)=\frac{P_{pred}}{P_{obs}}$$

$$Average\;fold\;error\;\left(AFE\right)=10^{\frac{\sum \log\left(FE\right)}{n}}$$

where *P_pred_* is the estimated PK parameters from the model prediction and *P_obs_* is the estimated PK parameters from the observed concentrations, and *n* is the number of samples.

The validated minimal PBPK models were applied for the multiple-dosing simulation. PK profiles for the multiple oral dosing of pyronaridine–artesunate with either low or high doses (180/60 or 360/120 mg/kg) once a day for 3 or 14 days were simulated in hamsters. An elimination half-life, accumulation ratio, time to reach a steady-state, and average lung, trachea, or blood concentration in the steady-state were evaluated using the simulated profiles of pyronaridine, artesunate, and dihydroartemisinin. The predictive performance of the minimal PBPK model was examined by comparing the observed and simulated concentrations in blood, lung, and trachea. The agreement of observed and simulated PK profiles was graphically evaluated.

### 2.4. Parameters Estimation

A noncompartmental analysis (NCA) was used to estimate the model-independent PK parameters. The elimination rate constant (*k_e_*) was estimated by linear regression analysis and the elimination half-life (t_1/2_) was divided ln 2 by *k_e_*. The maximum plasma concentration (*C_max_*) and time to maximum concentration (*T_max_*) were obtained by a visual observation of the plasma concentration time profiles. The area under the plasma concentration time curve from zero to time t (AUC_t_) was calculated using a linear trapezoidal method. The average plasma concentration at a steady state (*C_avg_*) in a repeated dosing simulation was calculated by dividing the AUC_τ_ by the dosing interval (τ). The accumulation ratio was calculated using the ratio of the AUC_0-24 hr_ at day 1 and AUC_τ_ at a steady-state in multiple-dosing PK profiles.

## 3. Results

### 3.1. Pharmacokinetics, Lung, and Tissue Distribution of Pyronaridine and Artesunate

Since pyronaridine was uptaken by red blood cells and showed a high blood-to-plasma ratio (4.9–17.8), whole blood was selected as the biological matrix for the quantification of pyronaridine [34]. The blood-to-plasma ratios of artesunate and dihydroartemisinin were reported to be 0.75, suggesting that plasma rather than whole blood is the preferred matrix [21]. Thus, the plasma concentrations (*C_plasma_*) of artesunate and dihydroartemisinin were first quantified, and the blood concentrations were calculated by multiplying *C_plasma_* by *K_b:p_*.

**Figure 2:**
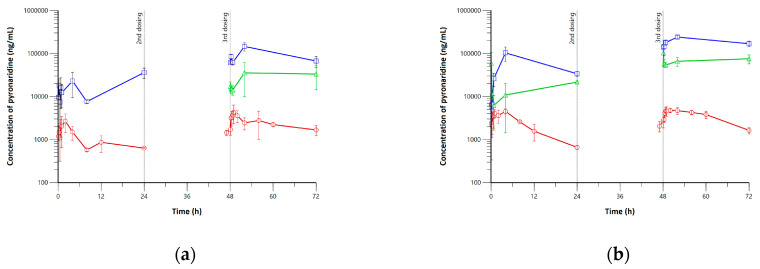
Pharmacokinetic profiles for (**a**) low-dose (180 mg/kg) and (**b**) high-dose (360 mg/kg) groups of pyronaridine in hamsters. Blue squares, green triangles, and red circles with solid lines represent lung, trachea, and blood concentrations of pyronaridine, respectively.

A naïve pooled-data approach was used for both NCA and PBPK modeling since the sparse sampling was required to obtain blood, plasma, lung, and trachea samples in hamsters [35]. The blood, lung, and trachea PK profiles after daily multiple dosing of pyronaridine (180 or 360 mg/kg) are shown in Figure 2. After the single dosing, the *T_max_* was estimated to be 2–4 h, and *C_max_* was calculated to be 2701.6 and 4542.4 ng/mL for the low- and high-dose groups, respectively. The calculated AUC_Day1_ was estimated to be 24,134.4 and 51,016.0 h·ng/mL for the low- and high-dose groups, respectively. The calculated AUC_Day3_ was estimated to be 57,084.6 and 84,867.9 h·ng/mL for the low- and high-dose groups, respectively. The lung and trachea exposures of pyronaridine were higher than blood exposures from the graphical evaluation for both low- and high-dose groups. The majority of pyronaridine was distributed to the lung and trachea with high average exposure ratios of 25.5 and 7.17 for the lung-to-blood AUC ratio (=AUC_lung_/AUC_blood_) and trachea-to-blood AUC ratio (=AUC_trachea_/AUC_blood_) on day one. Also, lung-to-blood and trachea-to-blood AUC ratios of pyronaridine were calculated to be 51.00 and 16.60 on day three.

**Figure 3:**
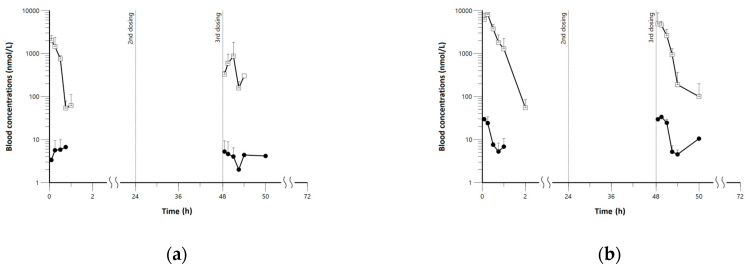
Blood concentrations for artesunate and dihydroartemisinin after multiple dosing of (**a**) low-dose (60 mg/kg) and (**b**) high-dose (120 mg/kg) artesunate to hamsters. Closed circles and open squares are blood concentrations of artesunate and dihydroartemisinin, respectively.

The observed blood concentrations of artesunate and dihydroartemisinin in the low- and high-dose groups are shown in Figure 3. An absorption phase of artesunate could not be obtained from all dose groups since artesunate immediately entered the systemic circulation. Also, both artesunate and dihydroartemisinin were all eliminated within about 2 h, and the accumulation was not caused by the dosing interval of 24 h. This result agrees with the previous report that describes peak concentrations being achieved rapidly (less than five minutes) for both compounds, and artesunate concentrations were decreased below the quantification limit in 2 h with no accumulation [36]. 

### 3.2. Development of Minimal Physiologically-Based Pharmacokinetic Models

For accurate predictions of systemic, lung, and trachea exposure, the minimal PBPK models were developed using the observed PK profiles of pyronaridine, artesunate, and dihydroartemisinin. The minimal PBPK models in this study were modified from Jermain et al., which reported the minimal PBPK model of ivermectin for COVID-19 drug repurposing [11]. Since the target organ for the antiviral effects of pyronaridine and artesunate were the lung and trachea, the nontarget tissues were lumped together into the rest of the body. The physiological parameters (*Q_co_*, *Q_trachea_*, *Q_rest_*, *V_blood_*, *V_lung_*, *V_trachea_*, and *V_rest_*) were fixed from the literature or measured from animal experiments. Generally, each tissue compartment in the PBPK model is described by either perfusion rate-limited kinetics or permeability rate-limited kinetics. In the perfusion rate-limited kinetics, it is assumed that the drug immediately across the membranes and tissue-to-blood concentrations in equilibrium are determined by the *K_p_* value (typically for small lipophilic molecules) [37]. Contrarily, if the permeability becomes the rate-limiting process (e.g., large polar molecules), the tissue compartment should be divided into extracellular and intracellular spaces, that are separated by a diffusional barrier [38]. In the European Medicines Agency (EMA) assessment report, artesunate and dihydroartemisinin were reported to follow perfusion rate-limited kinetics with a high extraction ratio [39]. Furthermore, since pyronaridine is a lipophilic compound that represents slow elimination and extensive distribution, the perfusion rate-limited kinetics were used for developing the minimal PBPK models [40]. According to the EMA report, artesunate is rapidly metabolized to dihydroartemisinin by blood esterases. Thus, artesunate is generally considered a prodrug of dihydroartemisinin [36,39]. Also, artesunate was reported to be almost completely metabolized to dihydroartemisinin in vivo [41]. Therefore, artesunate was modeled to be thoroughly converted to dihydroartemisinin in the blood compartment by the CL/F, and the rate of dihydroartemisinin produced was described by multiplying the artesunate concentration and CL/F.

The biochemical parameters of pyronaridine, artesunate, and dihydroartemisinin were summarized in Table 2. The lung-to-blood partition coefficient (*K_lung_*) and trachea-to-blood partition coefficient (*K_trachea_*) were estimated to be 26.06 and 8.67 for pyronaridine, respectively. These values were similar to the tissue-to-blood AUC ratio (=AUC_tissue_/AUC_blood_) calculated from the observed PK profiles of pyronaridine on day one. The *K_lung_* and *K_lung,m_* for artesunate and dihydroartemisinin were estimated to be 10.33 and 0.34, respectively. Although not higher than pyronaridine, artesunate showed high lung distribution while dihydroartemisinin was less distributed in the lung than that in the blood (*K_lung,m_* < 1). The *K_trachea_* and *K_trachea,m_* for artesunate and dihydroartemisinin were estimated to be 1.48 and 1.08, respectively.

**Figure 4:**
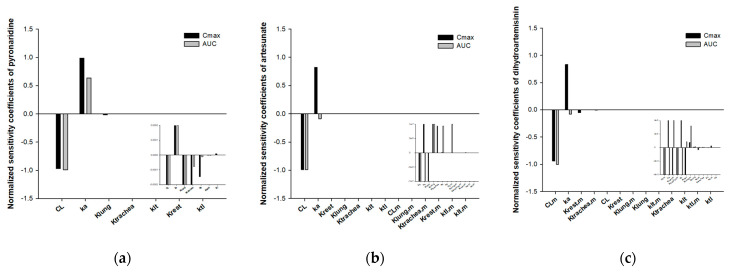
Normalized sensitivity coefficients for the minimal PBPK models of pyronaridine and artesunate. (**a**) pyronaridine; (**b**) artesunate; (**c**) dihydroartemisinin.

Also, the results of the sensitivity analysis (Figure 4) showed that normalized sensitivity coefficients for all parameters were within one, and most parameters were close to zero. Thus, the input error was considered to be not significantly amplified in the model output.

**Figure 5:**
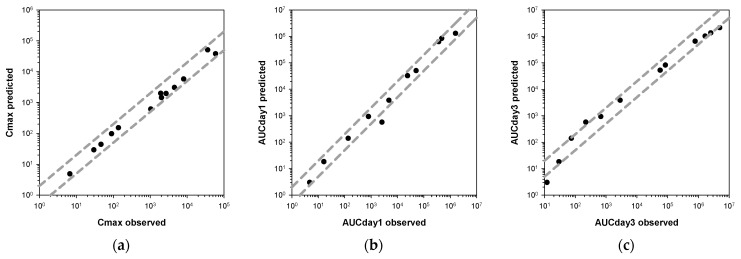
Comparison of the observed and predicted PK parameters. (**a**) C_max_; (**b**) AUC_Day1_; (**c**) AUC_Day3_. The dashed lines represent 2-fold errors between the observations and predictions.

The fold errors (FEs) for C_max_, AUC_Day1_, and AUC_Day3_ for the three compounds were calculated to evaluate the accuracy of the model predictions (Figure 5). The percentage of PK parameters that were within a twofold error was 100% for *C_max_*, 91% for AUC_Day1_, and 75% for AUC_Day3_, respectively. The average fold errors (AFEs) for *C_max_*, AUC_Day1_, and AUC_Day3_ were calculated to be 0.84, 0.94, and 0.86, respectively. Most of the FEs and all of the AFEs for PK parameters were under the twofold limit, suggesting the good predictive performances of the minimal PBPK models.

### 3.3. Application of Minimal Physiologically-Based Pharmacokinetic Models

Using the developed minimal PBPK models of pyronaridine and artesunate, the multiple-dosing simulations for low and high doses were conducted. Table 3 summarizes the PK parameters of pyronaridine, artesunate, and dihydroartemisinin estimated from the simulated PK profiles.

**Figure 6:**
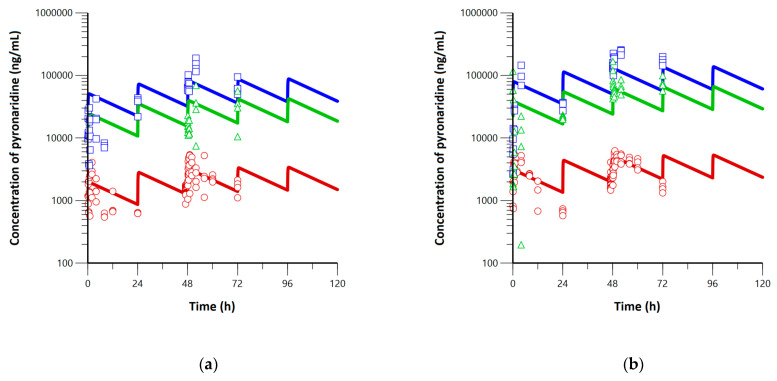
Simulated PK profiles for daily oral dosing of pyronaridine with (**a**) low-dose (180 mg/kg) and (**b**) high-dose (360 mg/kg) for 14 days. Blue, green, and red solid lines are simulated profiles for lung, trachea, and blood, respectively. Blue squares, green triangles, and red dots represent observed concentrations for lung, trachea, and blood, respectively.

The simulated PK profiles for pyronaridine were represented together with observed concentrations in Figure 6. In the simulated profiles of pyronaridine, the t_1/2_ was estimated to be 19.7–19.9 h. The elimination half-life can be used for the prediction of the drug accumulation and time to reach steady-state equilibrium [42]. The time to reach the steady state is dependent only on the elimination half-life [43,44]. The pharmacological rule states the steady state is achieved after four to five times of the elimination half-life [42,45,46]. Thus, since the t_1/2_ of pyronaridine was calculated to be 19.7–19.9 h, the steady state can be reached in about three to four days (79–100 h) after the first dosing. The elimination half-life of pyronaridine has not been reported in hamsters, while it has been reported to be two to four days in rats and 2.5 days in dogs, respectively [39]. The blood AUC_Day1_ was calculated to be 32,508.2 and 50,672.1 hr·ng/mL for the low- and high-dose groups, and the blood AUC_τ_ in the steady state was calculated to be 57,189.7 and 89,639.9 for the low- and high-dose groups, respectively. The accumulation ratio of pyronaridine was calculated to be 1.8 by dividing AUC_τ_ by AUC_Day1_. The mean blood concentrations at the steady state (*C_avg,blood_*) for the low- and high-dose groups were estimated to be 2382.9 and 3735.0 ng/mL. The *C_avg_* in the lung for the low- and high-dose groups were estimated to be 61,547.4 and 96,472.7 ng/mL. The *C_avg_* in the trachea for the low- and high-dose groups were estimated to be 29,579.9 and 46,366.1 ng/mL. At the steady state, the lung-to-blood concentration ratio (=*C_avg,lung_*/*C_avg,blood_*) and trachea-to-blood concentration ratio (=*C_avg,trachea_*/*C_avg,blood_*) were calculated to be 25.83 and 12.41 in all groups.

**Figure 7:**
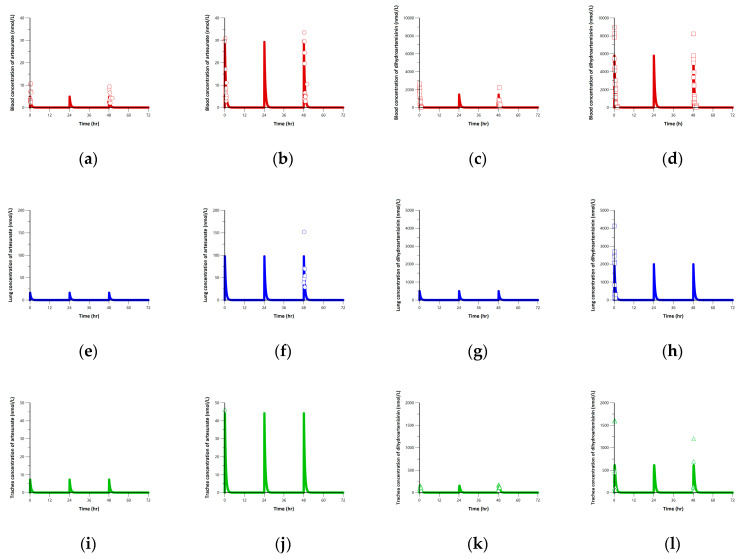
Simulated PK profiles for daily oral dosing of artesunate with low- and high-dose for 3 days. Blue, green, and red solid lines are simulated profiles for lung, trachea, and blood, respectively. Circles and squares are observed concentrations of artesunate and dihydroartemisinin, respectively. Blood concentrations of artesunate in low-dose (**a**) and high-dose (**b**) group; blood concentrations of dihydroartemisinin in low-dose (**c**) and high-dose (**d**) group; lung concentrations of artesunate in low-dose (**e**) and high-dose (**f**) group; lung concentrations of dihydroartemisinin in low-dose (**g**) and high-dose (**h**) group; trachea concentrations of artesunate in low-dose (**i**) and high-dose (**j**) group; trachea concentrations of dihydroartemisinin in low-dose (**k**) and high-dose (**l**) group.

The simulated PK profiles for artesunate and dihydroartemisinin are shown in Figure 7. Since the t_1/2_ of artesunate and dihydroartemisinin were all estimated to be 0.4 h, both compounds were eliminated within 2–3 h after dosing. This short elimination half-life suggests that the steady state is not achieved by daily multiple-dosing of artesunate. Therefore, the accumulation ratios of artesunate and dihydroartemisinin were all calculated to be one. The EMA report showed the elimination half-life for both artesunate and dihydroartemisinin was similar, ranging from 0.32 to 0.52 h [36]. In another report, only the PK parameters for dihydroartemisinin could be determined since artesunate is rapidly metabolized to dihydroartemisinin in vivo [39]. The reported elimination half-life of dihydroartemisinin was ranged from 0.25 to 1.03 h in rats and from 0.39 to 0.66 h in dogs [36,47]. The AUC_t_ of artesunate was calculated to be 3.0 and 18.3 h·nmol/L for the low- and high-dose groups, and that of dihydroartemisinin was calculated to be 931.4 and 3849.7 h·nmol/L for each group. Since artesunate and dihydroartemisinin were not accumulated by dosing intervals of 24 h, the steady-state could not be reached. Thus, the tissue-to-blood AUC was used for evaluating the tissue exposures instead of the *C_avg_* at the steady state. The lung-to-blood AUC ratios for artesunate and dihydroartemisinin were calculated to be 3.34 and 0.34. The trachea-to-blood AUC ratios for artesunate and dihydroartemisinin were calculated to be 1.51 and 0.15.

A minimum toxic concentration (MTC) or minimum effective concentration (MEC) of pyronaridine and artesunate has not been reported in hamsters. Nevertheless, a maximal nonlethal dose of pyronaridine and artesunate was reported to be 1500 and 500 mg/kg in rodents [39]. In addition, artesunate appeared to be more toxic than pyronaridine whether it was administered alone or coadministered with pyronaridine [39]. Thus, since the doses of both drugs used in this study were about four to eight times lower than the lethal dose and unscheduled deaths of the animals were not observed, the doses were regarded as tolerated. Also, several studies reported the antiviral effects against SARS-CoV-2 for pyronaridine and artesunate in vitro. In the case of pyronaridine, Bae et al. showed pyronaridine could suppress the replication of SARS-CoV-2 in Vero cells, and the EC_50_ was calculated to be 569.9 and 1139.7 ng/mL after 24 and 48 h of culture, respectively [9]. Gendrot et al. reported that the EC_50_ for an effective anti-SARS-CoV-2 was 373.0 ng/mL in Vero E6 cells [48]. Aherfi et al. showed the anti-SARS-CoV-2 activity of pyronaridine at a concentration of 103.6 (Huh7.5 cells) and 8.6 ng/mL (Calu-3 cells) [49]. In the case of artesunate, Zhou et al. proved the range of the EC_50_ for artesunate was 18.2–31.2 μM in different cell types [50]. Cao et al. reported similar anti-SARS-CoV-2 effects of artesunate and dihydroartemisinin with EC_50_ values of 12.98 and 13.31 μM, respectively [8].

## 4. Conclusions

The pharmacokinetics, trachea, and lung exposures of pyronaridine, artesunate, and dihydroartemisinin were successfully evaluated in hamsters using NCA and minimal PBPK modeling. The majority of pyronaridine was distributed to the lung and trachea with high average exposure ratios of 25.5 and 7.17 for the lung-to-blood and trachea-to-blood AUC ratio on day one. Also, the lung-to-blood and trachea-to-blood AUC ratios of pyronaridine were calculated to be 51.00 and 16.60 on day three. Artesunate and dihydroartemisinin were all eliminated within about 2 h and not accumulated by the dosing interval of 24 h. Using the minimal PBPK model, the multiple-dosing simulations for daily oral dosing of pyronaridine and artesunate were conducted for 14 days and 3 days, respectively. The steady-state was reached about three to four days after the first dosing of pyronaridine, and pyronaridine was extensively distributed to the lung and trachea with the lung-to-blood and trachea-to-blood concentration ratios of 25.83 and 12.41, respectively. Artesunate and dihydroartemisinin were eliminated within 2–3 h after dosing, suggesting the steady-state is not achieved by a daily multiple-dosing of artesunate. Nevertheless, the lung-to-blood AUC ratios for artesunate and dihydroartemisinin were calculated to be 3.34 and 0.34 from the single-dose PK profiles, respectively. Also, the trachea-to-blood AUC ratios for artesunate and dihydroartemisinin were calculated to be 1.51 and 0.15 from the single-dose PK profiles, respectively. The results of this study could be used as a scientific basis for establishing the correlation between blood, lung, or trachea exposures with antiviral activity against SARS-CoV-2 of pyronaridine and artesunate.

## Figures and Tables

**Table 1:** Physiological parameters for developing the minimal PBPK model.

Parameter	Unit	Description	Value	Reference
*V_total_*	mL	Total body volume	102.01	Experimental data
*V_blood_*	mL	Blood volume	7.20	[22]
*V_lung_*	mL	Lung volume	0.48	Experimental data
*V_trachea_*	mL	Trachea volume	0.06	Experimental data
*V_rest of body_*	mL	Volume of the rest of the body	94.27	Calculated ^1^
*Q_co_*	mL/hr	Cardiac output	1181.28	[23]
*Q_trachea_*	mL/hr	Blood flow rate for the trachea	24.81	[24,25,26]
*Q_rest_*	mL/hr	Blood flow rate for the rest of the body	1156.47	Calculated ^2^
*K_b:p_*	-	Blood-to-plasma partition coefficient (artesunate)	0.75	[21]
*K_b:p,m_*	-	Blood-to-plasma partition coefficient (dihydroartemisinin)	0.75	[21]

^1^ *V_total_* − (*V_blood_* + *V_lung_* + *V_trachea_*); ^2^ *Q_co_* − *Q_trachea_*.

**Table 2:** Biochemical parameters of pyronaridine, artesunate, and dihydroartemisinin.

Parameter	Unit	Value
Pyronaridine		
k_a_	1/h	0.03
CL/F	L/h	0.21
*K_lung_*	-	26.06
*K_trachea_*	-	8.67
*K_rest_*	-	5.25 × 10^−7^
*k_tl_*	1/h	1.01
*k_lt_*	1/h	0.92
Artesunate and dihydroartemisinin		
k_a_	1/h	1.74
CL/F	L/h	2517.70
*K_lung_*	-	10.33
*K_trachea_*	-	1.48
*K_rest_*	-	1.32
*k_tl_*	1/h	1.50
*k_lt_*	1/h	0.34
CL_m_/F	L/h	10.33
*K_lung,m_*	-	0.34
*K_trachea,m_*	-	1.08
*K_rest,m_*	-	1.21
*k_tl,m_*	1/h	6.98
*k_lt,m_*	1/h	0.35

**Table 3:** Pharmacokinetic parameters of pyronaridine, artesunate, and dihydroartemisinin estimated from the simulated multiple-dosing PK profiles.

Parameters	Pyronaridine		Artesunate		Dihydroartemisinin	
	Day 1	Steady-State	Day 1	Steady-State	Day 1	Steady-State
Low-dose group						
*C_avg,blood_* ^1^	1354.5	2382.9	0.1	0.1	38.8	38.8
*C_avg,lung_* ^1^	34,978.8	61,547.4	0.4	0.4	13.4	13.4
*C_avg,trachea_* ^1^	16,806.7	29,579.9	0.2	0.2	5.9	5.9
AUC_blood_ ^2^	32,508.2	57,189.7	3.0	3.0	931.4	931.4
AUC_lung_ ^2^	839,491.5	1,477,137.6	10.1	10.1	320.5	320.5
AUC_trachea_ ^2^	403,360.1	709,917.6	4.6	4.6	140.5	140.5
t_1/2_ (h)	19.7		0.4		0.4	
Accumulation ratio	1.8		1		1	
High-dose group						
*C_avg,blood_* ^1^	2111.3	3735.0	0.8	0.8	160.4	160.4
*C_avg,lung_* ^1^	54,525.7	96,472.7	2.5	2.5	55.2	55.2
*C_avg,trachea_* ^1^	26,199.6	46,366.1	1.2	1.2	23.8	23.8
AUC_blood_ ^2^	50,672.1	89,639.9	18.3	18.3	3849.7	3849.7
AUC_lung_ ^2^	1,308,615.7	2,315,344.4	61.2	61.2	1324.9	1324.9
AUC_trachea_ ^2^	628,789.5	1,112,786.7	27.6	27.6	570.5	570.5
t_1/2_ (h)	19.9		0.4		0.4	
Accumulation ratio	1.8		1		1	

^1^ ng/mL for pyronaridine; nmol/L for artesunate and dihydroartemisinin; ^2^ h·ng/mL for pyronaridine; h·nmol/L for artesunate and dihydroartemisinin; AUC_τ_ in multiple-dosing PK profiles.

## Data Availability

The data presented in this study are available on request from the corresponding author. The data are not publicly available due to privacy issues.
